# Promoting health and productivity among ageing workers: a longitudinal study on work ability, biological and cognitive age in modern workplaces (PROAGEING study)

**DOI:** 10.1186/s12889-023-16010-1

**Published:** 2023-06-12

**Authors:** Matteo Bonzini, Anna Comotti, Alice Fattori, Daniele Serra, Marco Laurino, Francesca Mastorci, Pasquale Bufano, Catalina Ciocan, Luca Ferrari, Valentina Bollati, Cristina Di Tecco

**Affiliations:** 1 Occupational Medicine Unit, Foundation IRCCS Ca’ Granda Ospedale Maggiore Policlinico, Milan, Italy; 2 Department of Clinical Science and Community Health, University of Milan, Milan, Italy; 3 School of Medicine, University of Milan, Milan, Italy; 4 Institute of Clinical Physiology, National Research Council, Pisa, Italy; 5 Department of Public Health and Pediatrics, University of Turin, Turin, Italy; 6 Department of Occupational and Environmental Medicine, Epidemiology and Hygiene, Italian Workers’ Compensation Authority (INAIL), Rome, Italy

**Keywords:** Ageing workers, Workability, Cognitive ability, Occupational stress, Sleep quality, Biomarkers, Psychosocial risk factors

## Abstract

**Background:**

Large changes in ageing population and in retirement age are increasing the number of older people in the workforce, raising many challenges for policymakers in promoting employment opportunities and health for older workers. In this respect, longitudinal assessments of workability, well-being perception and cognitive skills over time may allow to detect factors influencing workers’ health. Moreover, new available molecular markers permit the measurement of biological age and age-related changes. Most studies analysed one aspect at time (psychological, biological, labour productivity), without considering their interaction. Aims of the study are to evaluate the relationship between workability, cognitive skills, and biological age in a population of ageing workers; to conduct a cross-sectional analysis to assess the impact of occupational exposures on workability, cognitive skills, and biological age; to evaluate inter-individuals changes in a prospective analysis with a re-evaluation of each worker.

**Methods:**

Our study plans to enrol 1000 full-time workers, aged over 50, undergoing the medical surveillance required by the current Italian Legislation. Data collection includes information about: (a) work ability and psychosocial risk factors (work ability index, HSE Management Standard-21 item, Utrecht Work Engagement Scale, World Health Organisation-Five, Well-Being Index, job satisfaction, general well-being, technostress); (b) cognitive skills (Stroop Color and Word test, Simon task, Corsi’s block-tapping test, Digit span test); (c) sleep habits and psychological well-being (Pittsburgh Sleep Quality Index, Insomnia Severity Index, Ford Insomnia Response to Stress Test; Symptom Check List 90, Psychological Well-Being Index, Profile of Mood State, Beck Depression Inventory, Beck Anxiety Inventory, Perceived Stress Scale, Brief COPE); (d) biological age (telomere length, DNA methylation) for 500 workers. All workers will repeat the evaluation after one year.

**Discussion:**

This study aims to increase our knowledge about interactions between work ability, cognitive ability, well-being perception and psychological status also by including molecular markers, with a longitudinal and multidisciplinary approach. By bringing better insights into the relationship between risk factors and their impact on perceived and biological health, this study also aims at identifying possible interventions and protective measures to ensure aged workers’ well-being, consistent with all the eminent calls for actions promoted by key International and European labour organizations.

## Background

Ongoing transformations and innovations are changing almost every aspect of modern jobs and workplaces. The use of digital technologies has risen markedly, and employees are requested to rapidly update and face cognitive adaptation to changes. As an effect of the *24-Hour Society*, exposure to shift work and work outside office hours is increasing. Moreover, demographic changes entail the raising of the average age of the workforce and the average normal retirement: the average age of Italians constantly rose in the past years and Italy has now the second-oldest population, behind only Japan. [1]

These factors pose a significant challenge to occupational health and safety of aged workers (AW), i.e., those over 50 years old, especially if exposed to heavy workloads and risk factors to which they may be more vulnerable compared to younger workers. However, when adequately supported and protected, AW may represent a crucial resource to the workplace.

Sustainable employment requires that job demands should be tailored to workers’ health and skills. Keeping AW healthy and productive is a key goal of European labour policy [2]. Workers and Institutions should collaborate in creating health promotion interventions that support older employees. For instance, Finland has governmental programs and national measures, which directly address the situation of ageing workers and aim to improve job competence and workability [3].

To guarantee AWs’ well-being and productivity, identification of the effects of prolonged exposure to adverse working conditions and of successful interventions to slow cognitive aging and improve work productivity is necessary. In this respect, longitudinal assessments of workability, well-being perception and cognitive skills over time may allow to detect factors influencing workers’ health, linking these aspects with new available molecular markers that permit the measurement of biological age and age-related changes. Improving AWs’ health and safety is definitely a key objective of European social policy. Nevertheless, most studies analysed just one aspect at time (psychological, biological, labour productivity), without considering their interaction.

Our study claims to integrate these aspects and to jointly evaluate workability, psychological outcomes, cognitive indices, and molecular markers, with a multifactorial and multidisciplinary approach. Moreover, longitudinal data allow comparing worker’s status over time, quantifying aging trend in the active workforce and consequently evaluating the long-term effects of the changes and of the health promotion interventions.

Lastly, our study is performed in strict collaboration with the occupational physician’s health surveillance, a privileged setting for systematically monitoring the health status of the worker population.

In more detail, the specific aims of this study are to:

- Evaluate the effects of the changing world of work and of the cumulative exposure to shift work, particularly night shifts, on aged workers’well-being and quality of life, productivity, and cognitive skills, and biological age.
- Quantify the impact of health promotion interventions over time.

## Methods/design

Our primary research question is to investigate how to improve aging workers’ productivity and well-being through a prospective observational study on AWs’ workability, cognitive ability, psychological well-being and biological age.

The study has three aims:

- To evaluate the relationship between workability, cognitive skills, and biological age in a population of AWs.
- To conduct a cross-sectional analysis to assess the impact of occupational exposures (shift work, occupational stress, physical workload, smart working) on workability, cognitive skills, and biological age.
- To evaluate inter-individual changes in a prospective analysis with a re-evaluation of each worker.

The *primary endpoint* is the assessment of workability – measured using the Work Ability Index (WAI, [4]) – among exposed workers. WAI index is considered as a reliable instrument for monitoring workability in ageing population, employed in both intellectual and physical jobs [5]. The age-related decline in workability was proved to be more rapid in shift workers and associated with shorter telomeres length (i.e., increased biological age) [6].

*Secondary endpoints* are psychological well-being, cognitive skills, and biological age.

- Workers’ health status and well-being are measured through self-reported psychometric questionnaires. The Pittsburgh Sleep Quality Index (PSQI; [7]), the Insomnia Severity Index (ISI; [8]) and the Ford Insomnia Response to Stress Test (FIRST; [9]) to assess sleep quantity, quality, and habits. The Symptom Check List 90 (SCL-90; [10]), the Psychological Well-Being Index (PGWBI; [11]), the Profile of Mood State (POMS; [12]), the Beck Depression Inventory (BDI-II) and the Beck Anxiety Inventory (BAI) [13], Perceived Stress Scale (PSS; [14]) and Brief COPE to measure mental health, mood states and coping strategies [15].
- Cognitive skills are assessed through cognitive tests: the Stroop Color and Word test (SCWT; [16]) and Simon Task for cognitive flexibility and attention span [17]; the Corsi’s block-tapping test for visual-spatial short-term memory [18]; the Digital Span test for verbal short-term memory [19]. Repeated measures will permit to evaluate possible within-subjects differences and to evaluate factors associated with cognitive impairment.
- Telomere length and DNA methylation, considered some of the best biomarkers of ageing, are used to measure biological age [20, 21].

### Study design

The study design is observational and longitudinal, non-pharmacological.The study is conducted in workplaces where the Occupational Medicine Unit of IRCCS Ca’ Granda Ospedale Maggiore Policlinico Foundation, Milan, Italy (Unit 1) and the Occupational Medicine Unit of University of Turin, Turin, Italy (Unit 2) carry out the medical surveillance required by Italian Legislation in terms of occupational safety (i.e., Legislative Decree n.81/2008). To include different work environments, we enrol subjects employed in different working sectors (banking and finance, chemical industry, metal-mechanic industry).

Company’s Doctors (Occupational physicians) from Unit 1 and Unit 2 involve the Health and Safety Manager and Workers’ Health and Safety Representatives and invite workers to participate to the study during the health surveillance activity.

### Participants

Our study claims to include 1000 aged workers; assessment measures will be performed at enrolment point (T0) and at follow-up, after 12 months (T1). Demographic and occupational information are acquired by all subjects.

Inclusion criteria are: full-time workers, aged over 50, with job seniority of at least 10 years in their current workplace, who signed the informed-consent form; workers must be exposed to one of the following conditions: nigh-shifts or remote work. No exclusion criteria based on gender, ethnicity, or clinical characteristic are considered.

### Data acquisition

A) Recruitment, data collection and blood test
  The occupational Physician invites workers undergoing periodical health surveillance to participate to the study. Subjects who sign the written consent are enrolled and identified with a pseudo-anonymous code. Through the REDCap platform ([22]), the occupational physician collects workers’ socio-demographic and occupational information (age, gender, weight, height, occupational role, shift work, remote work), pack/years, BMI, work-related history, job seniority, exposure to risk factors, clinical condition, and current disease.
  For a total of 500 workers with scheduled occupational health surveillance that entails blood samples (for shift work or other potential occupational risk factors) we also collect 14 ml of venous blood (in EDTA tubes) to assess biological age (see point E), at T0 and after 12 months (time 1).
B) Workability and psychosocial risk factors
  Once the worker is enrolled, he/she is administered a list of questionnaires on workability and work-related stress. The Occupational Physicians enter workers’ responses directly into REDCap Platform installed on a tablet device.
  Questionnaires include WAI, HSE Management Standard-21 item [23]. Utrecht Work Engagement Scale (UWES-3; [24]), World Health Organisation-Five Well-Being Index (WHO-5; [25, 26]), job satisfaction [27], general well-being [28, 29], and technostress [30].
  The WAI is used in occupational health surveillance to evaluate employees’ work ability during health examinations and workplace surveys. It takes into account psychosocial and physical factors related to work, as well as the employee’s mental and physical resources and his/her health condition. The questionnaire covers seven dimensions: 1) the individual’s current work ability compared with their lifetime best; 2) work ability in relation to the demands of the job; 3) number of diagnosed illnesses; 4) estimated impairment due to diseases/illnesses or limiting conditions; 5) amount of sick leave during the past 12 months; 6) personal prognosis of work ability 2 years prior the study; 7) estimate of mental resources.
  The WAI is calculated by summing the seven dimensions ranging from 7 to 49 points. Total score of WAI is categorized as poor (7 to 27 scores), moderate (28 to 36 scores), good (37 to 43 scores) and excellent (44 to 49 scores).
C) Cognitive skills
  Once completed the questionnaires, workers undergo a cognitive assessment through the following cognitive tests, Stroop Color Task, Simon Task, Corsi Blocks test and Digit Span, implemented on a mobile tablet application. Workers have access to the tests using the pseudo-anonymous code and complete them on their own. The mobile application for tablet has been developed by researchers from Institute of Clinical Physiology, National Research Council, Pisa, Italy (Unit 3).
D) Well-being and sleep habits
  Questionnaires on psychological well-being and sleep quality are completed by subjects remotely and with an individual link sent by email, with a personal password and instructions given by the physician during the first evaluation, in order to obtain complete confidentiality of collected data and personal information.
E) Biological age
  Seven milliliters of whole blood will be collected into 2 EDTA tubes (7ml each) from each participant by venous phlebotomy and sent to EPIGET laboratory to perform automated purification of DNA by Qiacube (Qiagen).
  The DNA samples will be aliquoted at a concentration of 25 ng/µL for DNA methylation analysis (DNA methylation age –DNAmAge-, LINE-1 and CLOCK) and telomere length analysis.
  DNAmAge will be assessed by analyzing the methylation levels from specific CpG sites measured in five selected markers (ELOVL2, C1orf132 / MIR29B2C, FHL2, KLF14, TRIM59). For DNA methylation analysis DNA samples will be plated at a concentration of 25 ng/µL in plates of 96 wells each and treated with sodium bisulfite using the EZ-96 DNA Methylation-Gold™ Kit (Zymo Research; Irvine, CA, USA) following the manufacturer’s instructions. After elution, each DNA sample will be divided into 10-µL aliquots using the Microlab STAR Automated Liquid Handling Workstation (Hamilton Company; Reno, NV, USA). 10 µL of bisulfite-treated template DNA will be added with 25 µL of GoTaq Hot Start Green Master mix (Promega), 1 µL of forward primer (10 µM), and 1 µL of 5′ end- reverse primer (10 µM) to set up a 50 µL PCR reaction. Biological age (Y) will be calculated using the following algorithm: Y = 8,052 + 55,673 * ELOVL2 + 47,141 * FHL2 + 62,870 * KLF14–29.075 * MIR2B29B2C + 41.281 * TRIM59 [31–33].

### Variables

For all participants we collect (a) physiological, pathological, and occupational history data including age, gender, weight, height, occupational role, job seniority, pack-years, night shifts, disease, medications, possible abnormal blood test results, occupational exposures (noise, physical demands, chemical risk, high temperatures), remote work; (b) workability and psychosocial risk factors (WAI, HSE Management Standard 21 item. Utrecht Work Engagement Scale (UWES-3), job satisfaction, technostress); (c) psychological wellbeing and sleep quality (PSQI, ISI, FIRST, SCL-90, PGWBI, POMS, BDI-II, BAI, PSS, Brief COPE); (d) cognitive skills (Stroop Color Task, Simon Task, Digit Span, Corsi Blocks Test).

For half of participant, in addition, we measure DNA-methylation in peripheral white blood cells.

DNA methylation analysis of DNA in peripheral blood cells from specific CpG sites measured in five selected markers (ELOVL2, C1orf132 / MIR29B2C, FHL2, KLF14, TRIM59).

Gene specific DNA methylation analysis of CLOCK. Global genomic DNA methylation content estimated in long interspersed nuclear element-1 (LINE-1) repeated elements.

Telomere Length (TL) is measured using the quantitative real-time method which measures the relative TL by determining the ratio of telomere repeat copy number (T) to single copy gene (human beta globin -hbg) (S) copy number (T/S ratio) in experimental samples relative to a reference sample.

### Sample size calculation

The planned number of participants is 1000.

The sample size was calculated on the basis of primary hypothesis to reach expected differences between exposed and non-exposed groups with a statistical power of at least 90% and assuming a type I error of 5%.

For a total sample size of 1000 with equal groups (500) of exposed and non-exposed (to night shift work or to high stress – i.e. perceived stress above median) with a standard deviation of WAI index equal to 5, we calculated differences greater or equal to 1 with 90% power. Such differences were lower than expected, if compared to previous results obtained among shift workers and non-shift workers undergoing health surveillance in Unit 1 (difference in WAI score equal to 4).

For the secondary endpoint, sample size of 500 with equal groups (250) of exposed and non-exposed (e.g. WAI score less/greater than the median or cognitive indices less/greater than the median) and standard deviation of telomeres length equal to 0.3, is sufficient for reaching differences of 0.2 with 90% power. Such differences were lower than previous results obtain in similar population [6].

### Data management

Data are stored and handled at the Unit 1 and at the Unit 3. Pseudo-anonymized databases are merged using the unique participant ID. Only the team members access all data recorded in an electronic database.

Blood tubes are labelled to guarantee proper identification, collected and analysed by the Environmental Epigenetics Lab (Unit 4).

### Data analysis

We will preliminarily conduct exploratory data analysis on the total sample, according to qualitative or quantitative variables character.

To measure changes in workability, we will perform the linear regression model considering WAI scores as continuous dependent variable and measuring the effect of possible associated variables (work exposures, wellbeing, psychological wellbeing, perceived stress, sleep quality) and confounders (age, gender, job seniority, pack/years, BMI).

For each subject, we will compute one relative ageing index, calculated as the difference between the measured biological indicators and the mean value of the same age group. Multiple regression analysis will investigate a possible association between the relative ageing index and work exposures, individual characteristics, clinical history and personal habits. In addition, we will measure the association between workability and biological indices through correlation analysis or regression analysis.

Appropriate analysis for repeated measures will be performed to analyse changes after 12 months (follow-up). We will measure the effect of work exposures, remote work, and programs on active ageing on workability, cognitive skills and biological ageing.

### Data quality

Quality of our data is guaranteed by grounding our investigation on the occupational physician health surveillance, which maximizes data reliability and minimizes the risk of difficulty understanding and the risk of untrue or uncompleted answers. Moreover, REDCap platform allows real-time data entry validation, anonymous responses, and secure identification of responses from survey participants through the unique ID. Database with questionnaires results can be quickly developed, easily exported to data analysis packages and managed by researchers from different institutions.

Biological material is stored in EPIGET Lab (Unit 4) and analysed according to the best biotechnologist practices.

## Discussion

Social and technological transformations are considerably changing workplaces, thus posing a challenge to workers’ health and safety; this issue may be more severe for aged workers. So far, little is known about the impact of night-shifts or remote work and their interplay with occupational risk factors on workability, psychological and cognitive outcomes of aged workers. This study aims to increase our knowledge about these interactions also by including molecular markers, with a longitudinal and multidisciplinary approach.

We are aware that this study has potential limitations therefore we have outlined possible solutions. Voluntary participation may be associated with low participation rate and unreliable answers. To minimize these risks, the study will be set in companies where the Occupational Physicians from Unit 1 and Unit 2 are conducting the health surveillance activity, as their prolonged experiences as company doctors will facilitate the engagement of employees, the cooperation with company organization and the communication of the results. The intended number of participants equal to 1000 corresponds to a conservative estimate of eligible workers who undergo periodical health surveillance and already assumes an estimated participation rate equal to 0.75.

We are also aware that workers’ previous occupational exposures may have affected biological age and wellbeing; for this reason, we planned to investigate this information and include them in the analysis. As any evaluation based only on the current occupation without considering previous cumulative exposure may be incorrect, only workers employed for at least 10 years in their current company and with available data on their health history and exposures will be eligible. Besides, compete workers’ medical records will give additional information on health status, which may affect workability, cognitive skills and biological age. Data on health condition, medical history and other determinants will be used as potential confounders in the statistical analysis.

By bringing better insights into the relationship between risk factors and their impact on perceived and biological health, this study also aims at identifying possible interventions and protective measures to ensure aged workers’ well-being, consistent with all the eminent calls for action promoted by key International and European labour organizations [34–37].

## Data Availability

The datasets used and/or analysed during the current study are available from the corresponding author upon reasonable request.

## List of abbrevations

AW: Aged workers
BAI: Beck Anxiety Inventory
BDI: Beck Depression Inventory
FIRST: Ford Insomnia Response to Stress Test
ISI: Insomnia Severity Index
PGWBI: Psychological Well-Being Index
POMS: Profile of Mood States
PSQI: Pittsburgh Sleep Quality Index WHO-5
PSS: Perceived Stress Scale
SCL: Symptom Check List
SCWT: Stroop Color and Word test
UWES: Utrecht Work Engagement Scale
WAI: Work Ability Index
WHO: World Health Organisation

## Declarations

### Study status

The study is ongoing.

Ethical approval statement was obtained in June 2021.

Subjects’ enrolment and data collection began in December 2021. Planned closing date is in November 2023.

We already enrolled a total of 468 workers, equal to 47% of the planned 1000 workers.

A total of 49 workers already performed the T1 follow-up evaluation.
